# Phase II study of stereotactic body radiotherapy with hydrogel spacer for prostate cancer: acute toxicity and propensity score-matched comparison

**DOI:** 10.1186/s13014-021-01834-1

**Published:** 2021-06-12

**Authors:** Mami Ogita, Hideomi Yamashita, Yuki Nozawa, Sho Ozaki, Subaru Sawayanagi, Takeshi Ohta, Keiichi Nakagawa

**Affiliations:** Department of Radiology, The University of Tokyo Hospital, 7-3-1 Hongo, Bunkyoku, Tokyo 113-8655 Japan

**Keywords:** Prostate cancer, Radiation therapy, Stereotactic body radiotherapy, Toxicity, Hydrogel, Patient-reported outcomes

## Abstract

**Background:**

The efficacy of a hydrogel spacer in stereotactic body radiotherapy (SBRT) has not been clarified. We evaluated the safety and efficacy of SBRT in combination with a hydrogel spacer for prostate cancer.

**Methods:**

This is a prospective single-center, single-arm phase II study. Prostate cancer patients without lymph node or distant metastasis were eligible. All patients received a hydrogel spacer insertion, followed by SBRT of 36.25 Gy in 5 fractions with volumetric modulated arc therapy. The primary endpoint was physician-assessed acute gastrointestinal (GI) toxicity within 3 months. The secondary endpoints were physician-assessed acute genitourinary (GU) toxicity, patient-reported outcomes evaluated by the EPIC and FACT-P questionnaires, and dosimetric comparison. We used propensity score-matched analyses to compare patients with the hydrogel spacer with those without the spacer. The historical data of the control without a hydrogel spacer was obtained from our hospital’s electronic records.

**Results:**

Forty patients were enrolled between February 2017 and July 2018. A hydrogel spacer significantly reduced the dose to the rectum. Grade 2 acute GI and GU toxicity occurred in seven (18%) and 17 (44%) patients. The EPIC bowel and urinary summary score declined from the baseline to the first month (P < 0.01, < 0.01), yet it was still significantly lower in the third month (P < 0.01, P = 0.04). For propensity score-matched analyses, no significant differences in acute GI and GU toxicity were observed between the two groups. The EPIC bowel summary score was significantly better in the spacer group at 1 month (82.2 in the spacer group and 68.5 in the control group).

**Conclusions:**

SBRT with a hydrogel spacer had the dosimetric benefits of reducing the rectal doses. The use of the hydrogel spacer did not reduce physician-assessed acute toxicity, but it improved patient-reported acute bowel toxicity.

*Trial registration*: Trial registration: UMIN-CTR, UMIN000026213. Registered 19 February 2017, https://upload.umin.ac.jp/cgi-open-bin/ctr_e/ctr_view.cgi?recptno=R000029385.

## Background

Surgery and radiation therapy are the two major definitive treatment options for prostate cancer. The role of radiation therapy will become more important in the field of prostate cancer treatment as the population ages.

Conventionally fractionated (1.8–2 Gy per fraction) intensity-modulated radiotherapy (IMRT) has been the standard regimen [1]. Recently, moderate hypofractionation (2.4–4 Gy per fraction) and ultra-hypofractionation (> 5 Gy per fraction) have become the preferred treatment options. Several randomized trials have shown that moderately hypofractionated IMRT had a similar efficacy and toxicity to those of a conventionally fractionated regimen [2–5]. Stereotactic body radiotherapy (SBRT) delivers a larger daily dose in small fractions in combination with precise image guidance. Prospective studies have shown that the efficacy and toxicity of SBRT were similar to those of conventional fractionation [6–14].

The proportion of patients treated with SBRT has been increasing steadily [15]. The shorter treatment duration of SBRT can improve patient convenience and may reduce health-care costs as well [16]. Moreover, hypofractionation with a larger fractional dose might result in better tumor control due to a lower α/β ratio of prostate cancer [17]. However, the increase in toxicity is a concern because of the larger biological doses applied [18].

A hydrogel spacer is a medical device that is inserted into the perirectal space to separate the rectum from the prostate. The placement of the spacer is temporary, and it is gradually absorbed over 6–12 months. A phase III randomized study showed that the use of a hydrogel spacer reduced the rectal dose and late gastrointestinal (GI) toxicity in conventionally fractionated IMRT [19, 20]. In addition to the clinical benefit, hydrogel spacer use may provide an economical advantage by reducing toxicity-related expenditures [21, 22]. Although hydrogel spacers are widely used in radiation therapy of prostate cancer, the clinical data of a hydrogel spacer in SBRT is limited. Hence, we conducted a prospective phase II study to determine the efficacy and safety of SBRT combined with a hydrogel spacer in prostate cancer patients. In this report, we presented our primary results of acute GI and GU toxicity, patient-reported outcomes (PROs), and dosimetric comparison. A comparison was also performed between patients with and without hydrogel spacers by using propensity score-matched analysis.

## Methods

### Study design and patients

This study was designed as a prospective single-center, single-arm phase II study. Eligible patients were men with pathologically proven prostate cancer and an age range of 20–80 years. Exclusion criteria included clinically positive lymph nodes, distant metastasis, history of prostate cancer treatment except for androgen deprivation therapy (ADT) less than 1 year before SBRT, prior radiation therapy to the abdomen and/or pelvis, and inflammatory bowel disease. This study was approved by the institutional review board (P2016022). The trial was registered with UMIN-CTR 000026213.

### Procedures

A hydrogel spacer (SpaceOAR system; Boston Scientific, Marlborough, MA, USA) was inserted into the perirectal space between the prostate and rectum before the initiation of SBRT. The hydrogel was injected using a transperineal approach with transrectal ultrasound guidance under local anesthesia. The CT scan was taken just before the spacer procedure as a reference image. About 1 week after the hydrogel spacer placement, magnetic resonance imaging (MRI) and the planning CT were performed. Bowel preparation included an anti-flatulence diet and laxative. On the day of the image simulation and the day of treatment, each patient treated with a full bladder and empty rectum by receiving an enema.

### Treatment planning and radiation therapy

Target volume and risk organs were defined by planning CT scans using CT/MRI fusion. The clinical target volume (CTV) included the prostate for low-risk, the prostate and the proximal 1 cm of the seminal vesicles (SV) for intermediate-risk, and the prostate and 2 cm of the SV for high- and very high-risk patients. If the patients had SV invasion, the whole SV was included in the CTV. The risk classification was based on the National Comprehensive Cancer Network risk classification for prostate cancer [1]. The planning target volume (PTV) was created by expanding the CTV by 3 mm posteriorly and 5 mm in any other direction. In addition to the clinical treatment plan, the treatment plan using CT scans before the spacer insertion was made for each patient for the dosimetric comparison. The prescription dose was 36.25 Gy to 95% of the PTV in five fractions with 6MV single-arc volumetric modulated arc therapy with flattening filter-free beams with a minimal dose of 35.9 Gy (99% of prescription dose) and a maximal dose of 42 Gy. The dose constraints for organ at risk were rectum V36.25 Gy < 5%, 32.625 Gy < 11%, V29 Gy < 20%, V27.19 Gy < 25%, V18.13 Gy < 50%, bladder V36.25 Gy < 8%, V18.13 Gy < 40%, V37 Gy < 10 cc, femoral head maximal dose ≤ 20 Gy, small bowel maximal dose ≤ 20 Gy, sigmoid colon V30 Gy < 1 cc, and penile bulb V29.5 Gy < 50%. All patients received SBRT using a linear accelerator every other day, excluding weekends. Intermediate- or high-risk patients were allowed to receive ADT at the treating physician’s discretion.

### Follow-up

Patients were seen at 2 weeks, 1 month, and 3 months after completion of SBRT, and then every 3 months for the first 1 year and every 6 months thereafter. Toxicity assessments were performed at the baseline, after the spacer insertion, during SBRT, and at each follow-up visit. Prostate specific antigen (PSA) was collected at the baseline, 1 month, 3 months after SBRT, and then every 3 months for the first 1 year and every 6 months thereafter.

### Endpoints

The primary endpoint was physician-assessed acute GI toxicity within 3 months after the SBRT completion. The secondary endpoints were physician-assessed acute genitourinary (GU) toxicity, physician-assessed late GI and GU toxicity, PROs with international prostate symptom score (IPSS), expanded prostate cancer index composite (EPIC) and functional assessment of cancer therapy-prostate (FACT-P), the spacer placement success rate, adverse events related to the spacer insertion, biochemical progression-free survival (bPFS), and dosimetric comparison of the target volume and risk organs before and after the spacer insertion. Acute toxicity was defined as that appearing within 3 months after the SBRT. Physician-assessed acute toxicity was assessed at the baseline and 2 weeks, 1 month, and 3 months after SBRT by the Common Terminology Criteria for Adverse Events (CTCAE) v4. IPSS was assessed at the baseline and 2 weeks, 1 month, and 3 months after SBRT. The PROs excluding the IPSS were measured at the same time points except for that at 2 weeks after SBRT. In this analysis, we report acute toxicity, PROs, and dosimetric comparison.

### Statistical analysis

The rate of acute GI toxicity assessed retrospectively in our institution in 26 consecutive SBRT patients without a hydrogel spacer was at 54%. A previous randomized controlled study on conventional fractionated IMRT reported a reduction in GI toxicity by 15% with the use of a hydrogel spacer [19]. Because SBRT patients have a higher incidence of acute toxicity than those with a conventionally fractionated IMRT regimen, a more significant impact of spacer use on toxicity reduction could be obtained [18]. We estimated that the spacer use would provide a 30% reduction in acute GI toxicity from 54 to 37%. Assuming an adverse event rate of 37% and the upper limit of the 90% confidence interval not exceeding a threshold of 54%, we calculated that 22 cases would be required. Considering the dropout, the target number of cases in this study was set at 25 cases. In December 2017, because of a favorable accrual and to increase the power, the target number of cases was increased to 40 cases. The paired T-test was used for the dosimetric comparison. The time course of the IPSS, EPIC, and FACT-P were assessed by one-way repeated measures analysis of variance (ANOVA).

A preplanned comparison was performed using the data of the retrospective cohort who received SBRT without a hydrogel spacer in our institution. Propensity score-matched analysis with a ratio of 1:1 was used to adjust the bias between patients with and without a spacer. Our retrospective cohort included 191 prostate cancer patients who received SBRT of 36.25 Gy in five fractions without a hydrogel spacer from May 2016 to February 2019. Only participants in this clinical study have received the hydrogel spacer until June 2018, because the hydrogel spacer was not available in Japan. We started using the hydrogel spacer routinely from May 2019 as a clinical practice. The same quality of life questionnaires were used in the retrospective cohort. Patients in the retrospective cohort were basically followed at 1 month, 3 months after SBRT and then every 3–6 months thereafter. The matching covariate included age, performance status, risk group, concurrent androgen deprivation therapy, anti-coagulation or platelet treatment, diabetes, and baseline IPSS score. After the propensity score matching, the rate of acute toxicity was assessed by the chi-square test. IPSS, EPIC and FACT-P scores of each time point were compared using the T-test and two-way repeated ANOVA. The T-test was used for dosimetric comparison. The statistical analyses were performed using the SPSS ver.24 (IBM Corporation, Armonk, NY, USA).

## Results

### Baseline characteristics

Between February 2017 and July 2018, forty patients were enrolled. Baseline characteristics are shown in Table 1. The median age of patients was 70 years (range 55–79). The majority of patients (63%) had intermediate-risk prostate cancer, while 30% had high- or very high-risk prostate cancer. One patient had a history trans-urethral resection of the prostate before SBRT.

**Table 1:** Patient baseline characteristics

	Spacer (n = 40)	
	n	%
*Age, years*		
Median (range)	70 (55–79)	
*BMI, kg/m*^*2*^		
Median (range)	23.8 (19.7–31.2)	
*Performance status*		
0	16	40
1	24	60
*Pre-treatment PSA, ng/mL*		
Median (range)	8.6 (2.3–195)	
≤ 10	25	62.5
10–20	11	27.5
20>	4	10
*Gleason score*		
6	4	10
7	24	60
8	6	15
9	6	15
*Clinical T stage*		
T1c	8	20
T2a	19	47.5
T2b	1	2.5
T2c	9	22.5
T3a	2	5
T3b	1	2.5
*Risk group*		
Low	3	7.5
Intermediate	25	62.5
High	6	15
Very high	6	15
*Androgen deprivation therapy*		
Yes	23	57.5
No	17	42.5
*PSA at RT initiation, ng/mL*		
Median (range)	3.5 (0.02–16.3)	
*Anti-coagulation or platelet treatment*		
Yes	6	15
No	34	85
*History of abdominal surgery*		
Yes	9	22.5
No	31	77.5
*Diabetes*		
Yes	5	12.5
No	35	87.5
*Smoking*		
Never	12	30
Past	26	65
Current	2	5

*BMI* body mass index, *PSA* prostate-specific antigen, *RT* radiation therapy

### Procedure-related outcome

The hydrogel spacer placement was successful in 39 cases out of 40 cases (98%) as one patient failed to receive enough hydrogel because of the needle clogging due to an unintentional interruption in the injection. Severe adverse events related to the hydrogel spacer procedure were not observed. No grade 3 or higher procedure related adverse event was observed. One patient (2%) developed grade 2 prostatitis and seminal vesiculitis eight days after the procedure and was treated with oral antibiotics as an outpatient. Grade 1 adverse events occurred in 21 patients (53%); urinary tract pain in 8 patients, urinary frequency in 3 patients, hematuria in 3 patients, urinary retention in 2 patients, rectal pain (discomfort) in 4 patients, abdominal pain in 2 patients, and anal pain in 2 patients. All 8 cases of urinary tract pain occurred in 18 patients who received a urinary balloon catheter. After we stopped using a balloon catheter, no cases of urinary tract pain occurred. The cause of hematuria was urethral injury by the catheter in 2 patients and by the needle in 1 patient. These grade 1 adverse events occurred temporarily and improved spontaneously within a few days. Eighteen patients (45%) had no symptoms after the spacer insertion.

### Dosimetric comparison

**Fig. 1:**
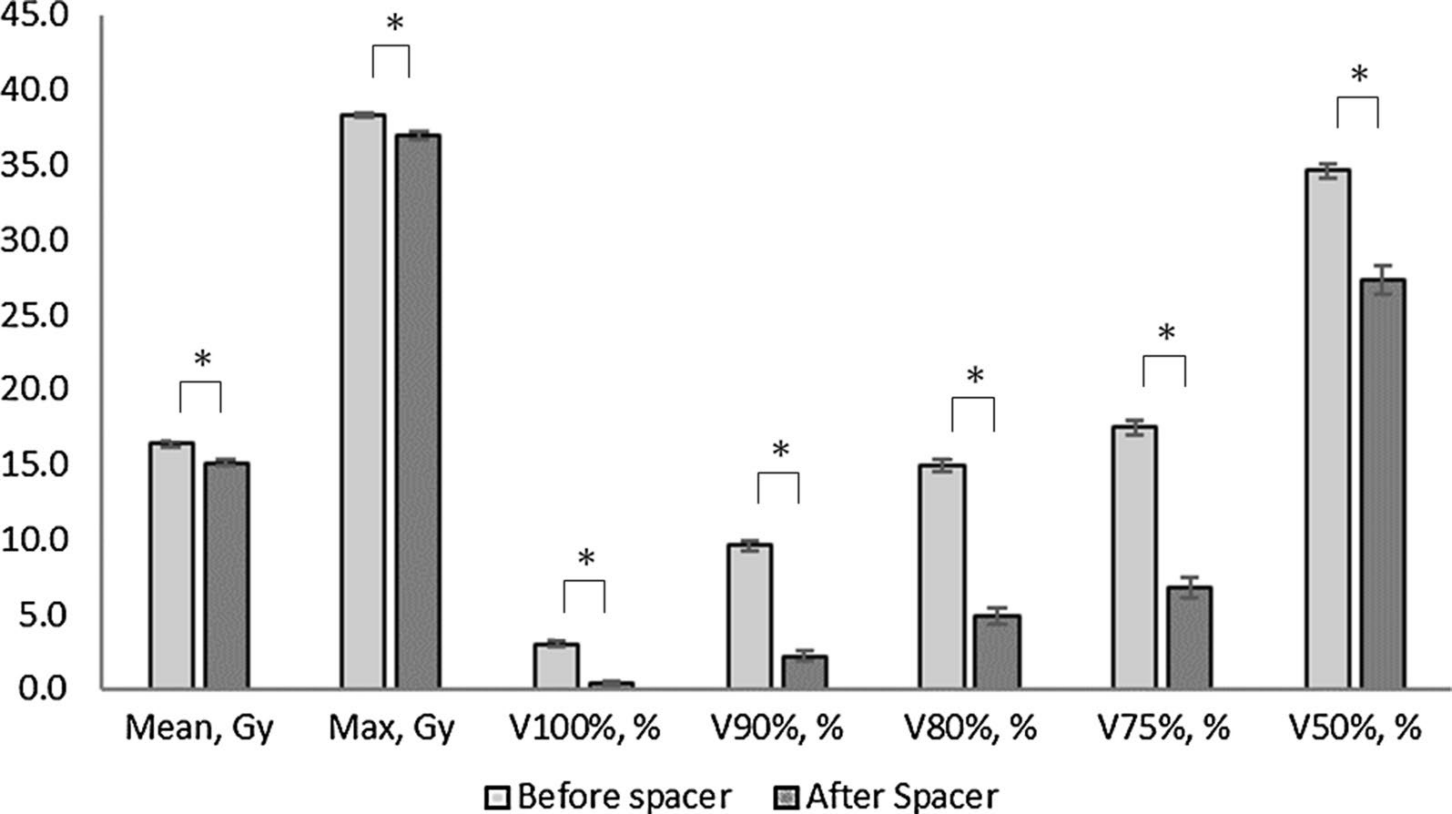
Dosimetric comparison of rectum doses before and after spacer insertion. * *P* < 0.01 comparison between before and after spacer insertion

Figure 1 and Table 2 show the dosimetric comparison before and after the spacer insertion. The rectal doses after the spacer insertion for the mean, maximal, and V100% to V50% were significantly lower compared to those before the spacer insertion (all *P* < 0.01). The means for rectal V100%, V90%, V80%, V75%, and V50% were reduced by 87%, 77%, 67%, 62%, and 21% with the spacer, respectively. The bladder mean, V50% and V100% doses, and maximal femoral head and penile bulb doses were significantly lower after the spacer insertion (femoral head *P* = 0.01, others *P* < 0.01). There were no differences observed in the mean and maximal PTV doses, and urethra and bladder maximal doses (*P* = 0.32, 0.20, 0.96 and 0.3, respectively).

**Table 2:** Dosimetric comparison of target and organs at risk before and after spacer insertion

	Before spacer (n = 40)	After spacer (n = 40)	*P*
	Mean (± S.D.)	Mean (± S.D.)	
Prostate, ml	42.2 (± 21.4)	39.6 (± 20.8)	< 0.01
Seminal vesicles, ml	13.6 (± 5.3)	13.2 (± 5.3)	0.34
PTV, ml	100.1 (± 36.2)	99.1 (± 36.7)	0.43
Bladder, ml	202.1 (± 102.8)	274.7 (± 132.2)	< 0.01
Rectum, ml	49.0 (± 13.0)	49.1 (± 22.2)	0.96
PTV mean, Gy	37.3 (± 0.3)	37.2 (± 0.3)	0.32
PTV max, Gy	39.1 (± 0.6)	39.0 (± 0.6)	0.20
Urethra max, Gy	38.3 (± 0.6)	38.3 (± 0.6)	0.96
Rectum mean, Gy	16.4 (± 1.2)	15.1 (± 1.4)	< 0.01
Rectum max, Gy	38.3 (± 0.6)	36.9 (± 1.8)	< 0.01
Rectum V100%, %	3.0 (± 1.2)	0.4 (± 0.6)	< 0.01
Rectum V90%, %	9.6 (± 2.2)	2.2 (± 2.2)	< 0.01
Rectum V80%, %	14.9 (± 2.7)	4.9 (± 3.5)	< 0.01
Rectum V75%, %	17.5 (± 2.9)	6.8 (± 4.1)	< 0.01
Rectum V50%, %	34.6 (± 3.1)	27.3 (± 5.9)	< 0.01
Bladder mean, Gy	12.4 (± 3.4)	10.6 (± 3.8)	< 0.01
Bladder max, Gy	38.7 (± 0.6)	38.5 (± 0.6)	0.30
Bladder V100%, %	4.8 (± 2.1)	3.8 (± 1.8)	< 0.01
Bladder V50%, %	26.2 (± 9.0)	22.6 (± 9.9)	< 0.01
Femoral head max, Gy	15.9 (± 1.9)	15.0 (± 1.4)	0.01
Penile bulb max, Gy	28.9 (± 8.1)	24.0 (± 10.6)	< 0.01

Rectal volume including the rectal contents

*PTV* planning target volume

### Physician-assessed acute toxicity and patient-reported outcomes

**Fig. 2:**
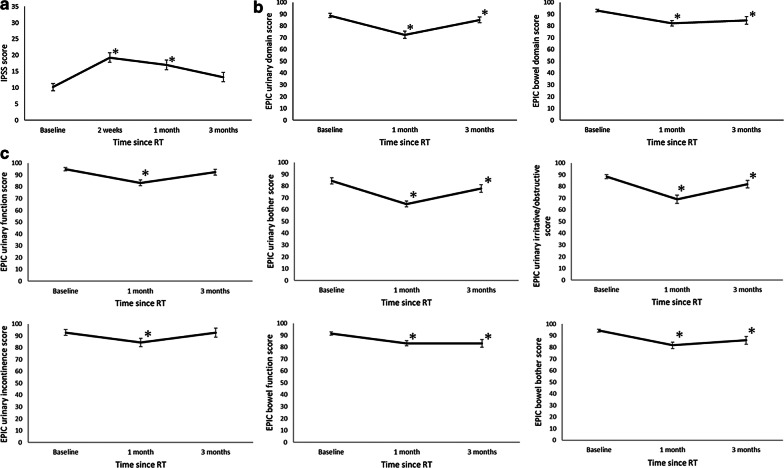
Time course of IPSS and patient-reported outcomes score **a** IPSS, **b** EPIC summary score, **c** EPIC subscale score. *P < 0.05 comparison between baseline and each time point. *IPSS* International Prostate Symptom Score, *EPIC* Expanded Prostate Cancer Index Composite

The grade 2 acute GI and GU toxicity was observed in seven (18%) and 17 (44%) patients, respectively (Table 3). No grade 3 or higher acute toxicity was observed. The mean IPSS temporarily increased at 2 weeks and 1 month after SBRT (*P* < 0.01, *P* < 0.01) and returned to baseline level in 3 months (*P* = 0.08) (Fig. 2a).

**Table 3:** Physician-assessed acute toxicity graded by the Common Terminology Criteria for Adverse Events (CTCAE)

	2w (n = 37)		1M (n = 39)		3M (n = 39)		Worst (n = 39)	
	n	%	n	%	n	%	n	%
*Acute gastrointestinal toxicity*								
Grade 0	17	46	11	28	19	49	5	13
1	17	46	25	64	17	44	27	69
2	3	8	3	8	3	8	7	18
*Acute genitourinary toxicity*								
Grade 0	1	3	2	5	4	10	0	0
1	28	76	27	69	31	80	22	56
2	8	22	10	26	4	10	17	44

Toxicity was graded according to the Common Terminology Criteria for Adverse Events (CTCAE) version 4

Higher values represent more favorable PROs in the EPIC and FACT-P scale. The EPIC scores are described in Fig. 2b, c. The EPIC urinary and bowel summary score declined from the baseline to the first month (*P* < 0.01, < 0.01), yet it was still significantly lower in the third month (*P* < 0.01, *P* = 0.04). For the urinary subscales, urinary bother and urinary irritative/obstructive declined from the baseline to the first and third month (*P* < 0.01*,* < 0.01) in the first month, and *P* = 0.02, 0.04 in the third month, respectively), while urinary function and urinary incontinence declined after 1 month and returned to baseline level in the third month (*P* < 0.01*,* < 0.01 in the first month, and *P* = 0.35, 0.63 in the third month, respectively). For the bowel subscales, both the bowel function and bother declined in the first month and third month from the baseline (all *P* < 0.01). In the FACT-P, physical well-being (PWB) significantly from the baseline to the first and third month (*P* < 0.01, *P* = 0.04). Prostate cancer subscale (PCS) significantly declined after 1 month (*P* < 0.01) and returned to baseline level in the third month (*P* = 0.41). The other FACT-P scales (social/family well-being (SWB), emotional well-being (EWB), functional well-being (FWB), FACT-G total, FACT-P total, and the trial outcome index (TOI)) did not show statistically significant changes.

### Propensity score-matched comparison

To balance the baseline differences, 39 patients with a spacer (spacer group) in this phase II study were matched with 39 patients who received SBRT without the spacer in our institution (control group). The patient characteristics and dosimetric comparison between two groups are shown in Tables 4, 5. The rectum doses and maximal bladder dose of the spacer group were significantly lower compared to those of the control group (all *P* < 0.01).

**Fig. 3:**
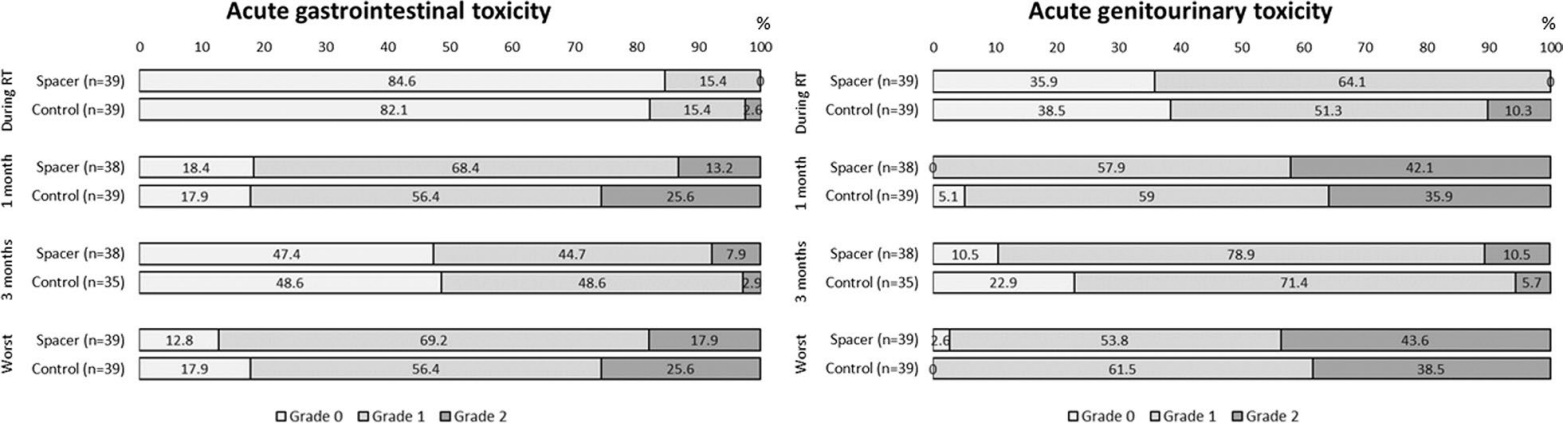
Comparison of acute gastrointestinal and genitourinary toxicity between the spacer group and the control group after propensity score-matching. Toxicity was graded according to the Common Terminology Criteria for Adverse Events (CTCAE) version 4

**Fig. 4:**
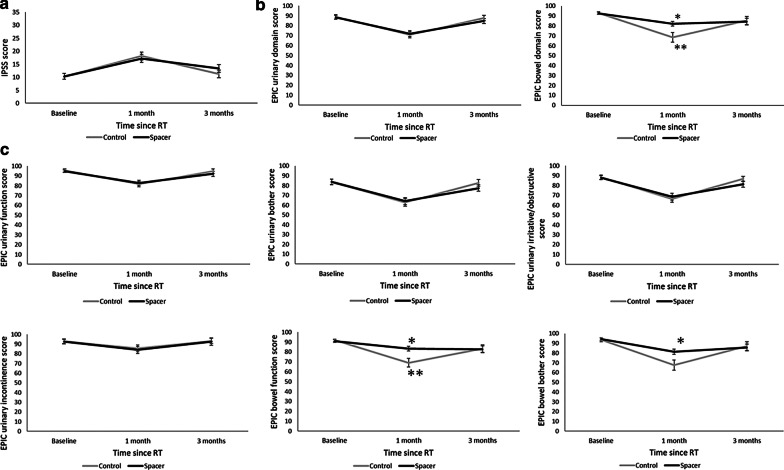
Time course of IPSS and patient-reported outcomes score **a** IPSS, **b** EPIC summary score, **c** EPIC subscale score after propensity score matching. *P < 0.05 comparison between the spacer group and the control group by T-test. **P < 0.05 comparison between the spacer group and the control group by two-way repeated ANOVA. *IPSS* International Prostate Symptom Score, *EPIC* Expanded Prostate Cancer Index Composite

There were no differences in acute GI and GU toxicity during RT, at 1 month, and at 3 months between the spacer and control groups (*P* = 0.60, 0.10 during RT, *P* = 0.37, 0.34 at 1 month, and *P* = 0.66, 0.31 at 3 months, respectively) (Fig. 3). Within 3 months after SBRT, 34 (87%) and 38 (97%) patients in the spacer group and 32 (82%) and 39 (100%) patients in the control group experienced GI and GU toxicity (*P* = 0.53, 0.31). Of them, the grade 2 or higher acute GI and GU toxicity was observed in 7 (18%) and 17 (44%) in the spacer group and 10 (26%) and 15 (39%) patients in the control group (*P* = 0.50, 0.51), respectively. There was no statistically significant difference in IPSS between the two groups (Fig. 4a). The EPIC urinary summary score and the subscales did not show statistically significant differences between the spacer and control groups (Fig. 4b, c). The EPIC bowel summary score was significantly higher in the spacer group at 1 month (82.2 in the spacer group and 68.5 in the control group, *P* < 0.01 with T-test and *P* = 0.02 with two-way ANOVA) (Fig. 4b). For the EPIC bowel subscale, bowel function and bowel bother scores were significantly higher in the spacer group at 1 month (83.4 and 81.5 in the spacer group and 69.1 and 67.7 in the control group, *P* < 0.01, *P* = 0.02 with T-test and *P* < 0.01, P = 0.07 with two-way ANOVA, respectively) (Fig. 4c). There were no differences in FACT-P scores between the two groups.

**Table 4:** Patient baseline characteristics between the spacer group and the control group after propensity score-matching

	Spacer (n = 39)		Control (n = 39)		*P*
	n	%	n	%	
*Age, years*					
Median (range)	71 (55–79)		69 (56–81)		0.87
*BMI, kg/m*^*2*^					
Median (range)	23.6 (19.7–29.7)		24.2 (20.4–38.2)		0.42
*Performance status*					
0	15	38.5	16	41	1.0
1	24	61.5	23	59	
*Pre-treatment PSA, ng/mL*					
Median (range)	8.3 (2.3–195)		8.6 (3.9–83.1)		
≤ 10	24	61.5	24	61.5	0.91
10–20	11	28.2	12	30.8	
20 >	4	10.3	3	7.7	
*Gleason score*					
6	3	7.7	5	12.8	0.36
7	24	61.5	28	71.8	
8	6	15.4	2	5.1	
9	6	15.4	4	10.3	
*Clinical T stage*					
T1c	8	20.5	13	33.3	0.49
T2a	18	46.2	18	46.2	
T2b	1	2.6	2	5.1	
T2c	9	23.1	4	10.3	
T3a	2	5.1	2	5.1	
T3b	1	2.6	0	0	
*Risk group*					
Low	2	5.1	0	0	0.4
Intermediate	25	64.1	30	76.9	
High	6	15.4	4	10.3	
Very high	6	15.4	5	12.8	
*Concurrent androgen deprivation therapy*					
Yes	23	59	21	53.8	0.82
No	16	41	18	46.2	
*PSA at RT initiation, ng/mL*					
Median (range)	3.4 (0.02–16.3)		3.6 (0.01–20.2)		0.78
*Anti-coagulation or platelet treatment*					
Yes	6	15.4	5	12.8	1
No	33	84.6	34	87.2	
*Diabetes*					
Yes	5	12.8	7	17.9	0.76
No	34	87.2	32	82.1	

*BMI* body mass index, *PSA* prostate-specific antigen, *RT* radiation therapy

**Table 5:** Dosimetric comparison of target and organs at risk between the spacer group and the control group after propensity score-matching

	Spacer (n = 39)	Control (n = 39)	*P*
	Mean (± S.D.)	Mean (± S.D.)	
Prostate, ml	39.2 (± 20.9)	39.3 (± 31.5)	1.00
Seminal vesicles, ml	13.1 (± 5.4)	10.1 (± 5.6)	0.02
PTV, ml	99.0 (± 37.2)	97.3 (± 49.2)	0.86
Rectum, ml	49.5 (± 22.3)	52.3 (± 15.8)	0.52
Bladder, ml	273.3 (± 133.6)	238.6 (± 119.3)	0.23
PTV mean, Gy	37.2 (± 0.3)	37.5 (± 0.4)	< 0.01
PTV max, Gy	39.0 (± 0.6)	39.5 (± 0.8)	< 0.01
Rectum mean, Gy	15.1 (± 1.3)	17.0 (± 1.3)	< 0.01
Rectum max, Gy	36.9 (± 1.8)	38.7 (± 0.7)	< 0.01
Rectum V100%, %	0.4 (± 0.7)	2.7 (± 1.6)	< 0.01
Rectum V90%, %	2.3 (± 2.2)	8.5 (± 2.7)	< 0.01
Rectum V80%, %	5.0 (± 3.5)	13.7 (± 3.4)	< 0.01
Rectum V75%, %	6.9 (± 4.1)	16.6 (± 3.8)	< 0.01
Rectum V50%, %	27.6 (± 5.6)	35.6 (± 5.2)	< 0.01
Bladder mean, Gy	10.7 (± 3.8)	12.2 (± 3.5)	0.09
Bladder max, Gy	38.6 (± 0.6)	39.1 (± 0.7)	< 0.01
Bladder V100%, %	3.8 (± 1.8)	4.2 (± 1.9)	0.39
Bladder V50%, %	22.9 (± 9.9)	25.2 (± 10.6)	0.32
Femur max, Gy	15.0 (± 1.5)	15.1 (± 1.8)	0.01

Rectal volume including the rectal contents

*PTV* planning target volume

## Discussion

Since the hydrogel spacer and SBRT are relatively new techniques and the reports on SBRT with a spacer are limited, we conducted a phase II study to evaluate the safety and efficacy of SBRT with a hydrogel spacer for prostate cancer. We reported the results of physician-assessed acute toxicity, PROs, and dosimetric comparison of SBRT with a hydrogel spacer for prostate cancer patients. Propensity score-matched analysis was also performed to compare with the retrospective cohort who received SBRT without a hydrogel spacer in our hospital.

Several studies and meta-analyses have evaluated the toxicity and oncologic outcomes of ultra-hypofractionation or SBRT versus conventionally or moderately fractionated IMRT for prostate cancer [9, 10, 18, 23, 24]. Ultra-hypofractionation or SBRT showed favorable tumor control and lower toxicity. There are two published randomized trials evaluating ultra-hypofractionation or SBRT comparing with conventional fractionation or moderately hypofractionation [13, 14]. A Scandinavian HYPO-RT-PC trial evaluated non-inferiority of ultra-hypofractionation of 42.7 Gy in seven fractions compared with the conventional fractionation of 78 Gy in 39 fractions [13]. The Radiation Therapy Oncology Group (RTOG) grade 2 or worse acute urinary toxicity was slightly higher in the ultra-hypofractionation group compared with conventional fractionation group (28% vs. 23%), but no difference was observed in RTOG grade 2 or worse bowel toxicity. PROs of both acute urinary and bowel symptoms within 3 months, evaluated by the PCSS questionnaire, were significantly higher in the ultra-hypofractionation group. PACE-B trial assessed the non-inferiority of SBRT (36.25 Gy in five fractions) compared with conventionally fractionated (78 Gy in 39 fractions) or moderately hypofractionated (62 Gy in 20 fractions) radiotherapy [14]. The acute RTOG grade 2 or higher toxicity was similar between SBRT and conventionally fractionated or moderately hypofractionated radiotherapy (10% versus 12% in GI toxicity (*P* = 0.38) and 23% versus 27% (*P* = 0.16) in GU toxicity, respectively). The acute CTCAE grade 2 or higher GI and GU toxicity rates were significantly higher in the SBRT group compared with conventionally fractionated or moderately hypofractionated radiotherapy (15.6% versus 9.1% (*P* < 0.01) in GI toxicity and 30.9% versus 23.7% (*P* < 0.01) in GU toxicity). There were no differences in EPIC scores between the two groups.

The results from randomized controlled trials suggest that the difference of acute toxicity between SBRT and conventionally fractionated radiotherapy varied according to the method of assessment. However, patients treated with SBRT tended to experience slightly higher acute GI and GU toxicity. PROs are presumably the most sensitive in detecting the acute toxicities followed by CTCAE, and RTOG.

A phase III randomized study showed that the use of a hydrogel spacer reduced the rectal dose and late GI toxicity, but there were no differences observed between acute GI and GU toxicity in conventionally fractionated IMRT [19, 20]. In our study, physician-reported acute GI and GU toxicity was similar in both the spacer and control groups. This result is consistent with the findings of the previous study. On the other hand, the use of a hydrogel spacer reduced the patient-reported acute bowel toxicity. Because acute toxicity of SBRT is slightly higher compared with that of conventionally fractionated IMRT, the reduction of acute bowel toxicity by a hydrogel spacer, which was not detected by the conventionally fractionated IMRT, was observed in our study.

The data from a combination of SBRT and spacer are limited. A retrospective study of 50 patients with low- and intermediate-risk prostate cancer analyzed the toxicity of SBRT with hydrogel spacer [25]. Acute GI and GU toxicity based on CTCAE occurred in 16% (grade 1) and 0% (grade 2), 30% (grade 1) and 34% (grade 2) during SBRT, and in 10% (grade 1) and 2% (grade 2), and 18% (grade 1) and 39% (grade 2) at 1 month post-SBRT, respectively. They did not compare with and without a hydrogel spacer, so the efficacy of the hydrogel spacer in SBRT was not shown in that study. A recently published study evaluated clinician-reported outcomes and PROs between the spacer and no-spacer patients as a subgroup analysis of the ongoing prospective observational study [26]. The results showed no difference between the pre- and post-SBRT in spacer and no-spacer groups, but the number of patients (10 patients in each group) may be too small to see a statistical difference.

The rectal dose was reduced by the spacer. These results are consistent with the findings of previous studies [19, 27, 28]. Bladder dose was also lower after the spacer insertion. Because the bladder volume was larger after the spacer insertion, the lower bladder dose would be due to the difference in bladder volume before and after the spacer insertion.

A hydrogel spacer could temporarily reduce acute bowel toxicity, but its effectiveness is limited as the cost of the spacer and procedure may not justify the use of the rectal spacers for all SBRT patients. A longer follow-up is necessary to clarify late toxicity.

Our study has several limitations, such as the relatively small sample size, the single institutional design, and the short follow-up duration. As this is a single-arm study, precise comparisons without a spacer cannot be made. Therefore, we conducted the propensity score-matched analysis using our retrospectively collected data from patients who received SBRT without the spacer in our institution. Although unknown confounders cannot be excluded, propensity score-matching can reduce the bias due to its confounding variables.

## Conclusions

A hydrogel spacer with SBRT had the dosimetric benefits of reducing the rectal doses. Although we did not show the significant reduction of physician-assessed toxicity, the use of a hydrogel spacer improved patient-reported acute bowel toxicity.

## Data Availability

The datasets used and/or analysed during the current study are available from the corresponding author on reasonable request.

## Abbreviations

ADT: Androgen deprivation therapy
ANOVA: Analysis of variance
bPFS: Biochemical progression-free survival
CTCAE: Common Terminology Criteria for Adverse Events
CTV: Clinical target volume
EWB: Emotional well-being
EPIC: Expanded prostate cancer index composite
FACT-P: Functional assessment of cancer therapy-prostate
FWB: Functional well-being
GI: Gastrointestinal
GU: Genitourinary
IMRT: Intensity-modulated radiotherapy
IPSS: International prostate symptom score
MRI: Magnetic resonance imaging
PCS: Prostate cancer subscale
PSA: Prostate specific antigen
PTV: Planning target volume
PWB: Physical well-being
PROs: Patient-reported outcomes
RTOG: Radiation Therapy Oncology Group
SBRT: Stereotactic body radiotherapy
SV: Seminal vesicles
SWB: Social/family well-being
TOI: Trial outcome index
